# Implementation protocol for a treatment bundle targeting modifiable risk factors for preterm birth

**DOI:** 10.1080/16549716.2025.2558366

**Published:** 2025-11-12

**Authors:** Gabrieli Roque de Castro, Bianca Milani Biazotto, Isabela Rodrigues da Silva, Gabriela Goes Herrera, Júlia Nepomucena Ruiz, Beatriz Marques Pizzinato, Karoline de Campos, Ana Lissa Camargo Pedroso, Alice Alves Gimenez, Nathália Lima Araújo Nunes, Gabriele Fernanda Oliveira Silva, Elis Marina Turini Claro, Natália Lucatto Vicaro, Ramkumar Menon, Rodolfo de Carvalho Pacagnella, Márcia Guimarães da Silva, João José Aguera Oliver Júnior, Bruna Ribeiro de Andrade Ramos

**Affiliations:** a Botucatu Medical School, São Paulo State University – UNESP, Botucatu, Brazil; b Jaú Medical School, São Paulo Western University – UNOESTE, Jaú, Brazil; c Department of Obstetrics and Gynecology, Division of Maternal-Fetal Medicine Perinatal Research, University of Texas Medical Branch, Galveston, TX, USA; d Department of Obstetrics and Gynecology of the Faculty of Medical Sciences of UNICAMP, Universidade Estadual de Campinas, Campinas, Brazil; e Ultra D Obstetrics and Gynecology Clinic, Jaú, Brazil

**Keywords:** Preterm birth, implementation research, prematurity prevention, vaginal dysbiosis, short cervix

## Abstract

Preterm birth (PTB) is the main cause of perinatal and neonatal morbidity and mortality worldwide. Widespread implementation of guidelines for early identification and management of patients at risk for adverse pregnancy outcomes is still feeble. This work aims to implement a simple and low-cost bundle to access and manage major modifiable risk factors for PTB. We included first trimester pregnant women seen at Health Units from Jaú – SP, Brazil, where PTB prevalence ranges from 13,4% to 18%. The protocol is based on three aspects: a questionnaire to access smoking status and clinical history; Gram staining and of vaginal microbiota evaluation using Nugent’s criteria and evaluation of cervical infections; and transvaginal ultrasound. Pregnant women who smoke and are willing to quit will be treated with auricular acupuncture and referred to a support group if necessary. All patients will be advised on intimate hygiene habits, and those with dysbiosis will be treated. Cervical length will be accessed using transvaginal ultrasound, and those diagnosed with cervical shortening will be treated with vaginal progesterone. This study highlights the importance of implementing active measures to reduce PTB risk factors in a high prevalence setting.

## Paper Context

- **Main findings:** This protocol suggests that addressing smoking, vaginal microbial imbalance, and cervical shortening may contribute to reducing the risk of preterm birth in high-prevalence populations.
- **Added knowledge:** The study adds evidence that integrating targeted interventions for modifiable biological and behavioral factors into routine prenatal care is both feasible and potentially effective.
- **Global health impact for policy and action:** The proposed strategy offers a scalable and low-cost approach that may help reduce preterm birth rates and related inequalities in maternal and child health, particularly in underserved regions.

## Background

Preterm birth (PTB), defined as birth before 37 weeks of gestation, is associated with perinatal morbidity and mortality worldwide. Preterm newborns are more likely to have short- and long-term complications, such as neurological impairment and increased susceptibility to infections, which can lead to irreversible sequelae and reduced quality of life [1].

Worldwide, the prevalence of PTB is approximately 11% [1], and Brazil is one of the most affected countries, with over 308,700 children born preterm every year, accounting for a preterm birth rate of 11.5%. In São Paulo state, 12% of births in 2023 were preterm, and a higher percentage was reported in the municipality of Jaú, where this adverse outcome affected 13.5% of all pregnancies [2].

PTB is a syndrome of multifactorial etiology with significant activation of inflammatory, oxidative stress, and senescence pathways [3]. PTB prevention should be a priority in public health policies; however, despite all the scientific and clinical efforts to fully elucidate and prevent PTB, protocols for early identification and effective management of women at risk have not yet been well-defined or effectively implemented, especially in Brazil. The main barrier to the implementation of such strategies is the gap in knowledge and communication between researchers, health professionals, and policymakers. In this context, three modifiable risk factors stand out for their well-established association with PTB: smoking habit, vaginal dysbiosis, and cervical shortening [4–9].

Smoking is a strong predictor of spontaneous preterm labor. Smoking during pregnancy has been associated with numerous adverse outcomes, including low birth weight, intrauterine growth restriction, prematurity, and infant mortality, due to heightened levels of inflammation and oxidative stress [4]. It is important to emphasize that smoking may be considered as a modifiable risk. In an epidemiological study based on national databases in the United States, Soneji et al. [4] observed that smoking cessation was associated with a relative reduction in the risk of PTB up to 23%, especially if the habit was stopped early in pregnancy. The authors also observed a dose-responsedose–response effect, with the risk of PTB being directly proportional to the number of cigarettes consumed. There are currently some strategies available to control smoking, such as pharmacological treatment, group support, and acupuncture. Studies suggest that auricular acupuncture can contribute to combating smoking through points that promote a reduction in anxiety and withdrawal symptoms [5].

Healthy vaginal microbiota is mainly colonized by *Lactobacillus* spp., which synthesizes lactic acid and other products that provide local protection against pathogens [6]. The replacement of the lactobacilli by anaerobic pathogens facilitates its ascent to the amniotic cavity and triggers an inflammatory response characterized by the production of cytokines and prostaglandins, ultimately inducing the labor-related events of myometrial contractility, cervical effacement, and rupture of the amniochorion membranes [7]. Although treatment for vaginal dysbiosis has not yet been proven effective in preventing PTB in late pregnancy, the condition is easily diagnosed by bacterioscopy, and the maintenance of eubiosis from the early stages of pregnancy may favor timely, adequate outcomes.

Another well-established risk factor for PTB is the length of the uterine cervix. A cervical length of less than 25 mm between the 16th and 24th gestational weeks is a strong independent predictor of spontaneous PTB. The risk of PTB in these cases is estimated at 25–30% in women with no history of PTB and up to 35% in pregnant women who have previously presented PTB. If the length is under 15 mm, the risk of PTB reaches 50%. In fact, several authors indicate the need for screening cervical length in this period via transvaginal ultrasound [8,9]. The management of cervical shortening (CS) includes the topical administration of progesterone, the use of pessary, and cerclage. A recent meta-analysis detected a significant reduction in the rate of extreme prematurity (< 33 weeks of gestation, < 2,500 grams birth weight) in pregnant women with CS (< 25 mm) who used intravaginal progesterone daily [9].

A common factor for the aforementioned conditions is the possibility of implementing preventive measures. Identifying risk factors for PTB early and before the development of irreversible clinical symptoms (e.g. cervical dilation, intraamniotic infection, oxidative damage associated inflammation) enables a personalized approach to patient care, aiming to reduce the risk of preterm birth and its sequelae. Thus, the primary aim of this study protocol is to reduce PTB in pregnant women from Health Units in the city of Jaú - SP, Brazil by implementing a low-cost bundle to assess and manage major modifiable risk factors for PTB. The secondary aim is to demonstrate the feasibility of our implementation protocol.

## Methods

### Study design

This is the protocol of an ongoing implementation study with a quasi-experimental controlled trial design conducted with pregnant women from primary health care units in the municipality of Jaú. The city of Jaú, located in the central region of the state of São Paulo, has a population of 151,881 inhabitants and 16 Basic Health Units (UBS) and Family Health Units (USF), in addition to a high-risk pregnancy clinic, Gestar, where this project is implemented.

**Figure 1.**
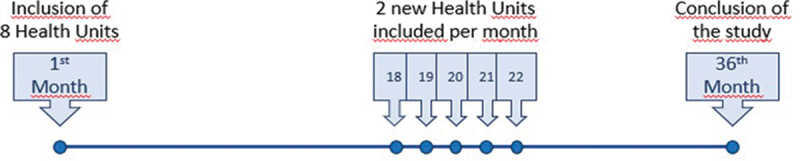
Temporal flowchart of cluster inclusion (health units) in the quasi-experimental design study.

The 16 UBS/USF were considered clusters and will be included in the study at different stages, according to the stepped wedge model, in which the clusters are gradually transferred to the intervention group at regular intervals until all have been transferred. This design is particularly useful in situations where the intervention cannot be implemented simultaneously for all units due to time, logistics, or ethical constraints [10]. In the first phase, eight of these clusters were randomly selected using a simple randomization process to receive the intervention, focusing on pregnant women during their first trimester. Over time, the remaining clusters will be randomly assigned to transition into the intervention group at regular intervals, using the same simple randomization method, ensuring that by the end of the study, all clusters will have received the intervention (Figure 1). This approach allows for a structured, phased implementation of the intervention, accommodating logistical and ethical constraints, and enables the comparison between clusters that have and have not yet received the intervention during the initial stages of the study.

During the first 18 months, all first-trimester pregnant women from eight randomized UBS/USF (clusters) are being included. In the next step of the study, 2 UBS/USF per month will be included until all primary care units (clusters) in the municipality are included, as demonstrated in Figure 1.

All pregnant women of eligible gestational age from the eight initially randomized health units were systematically invited to participate in the study and referred to Gestar. Cluster randomization was performed using a randomization tool (https://www.random.org/). Allocation was concealed and known exclusively by the coordinating researcher until implementation, minimizing the risk of selection bias. All participants included in the study will be monitored until the gestational outcome through three appointments, one in each gestational trimester. The inclusion criteria for the study are singleton pregnancy and gestational age between 11 weeks and 13 weeks and 6 days at the first appointment, defined by ultrasound. Pregnancies with fetal death and those attending fewer than two study appointments (first and second trimester) will be excluded from the study.

Considering the intervention aims to reduce the preterm birth rate from 18% (baseline rate, DataSUS) to 11.5% (São Paulo state rate = target rate), with 95% confidence, 5% margin of error, and 80% statistical power, the minimum sample size calculated is 201 patients (SAS 3.0 software). Adjusting the sample size to account for cluster effects (DE = 1+(m − 1)×ICC, where DE: design effect; mean cluster size: *m* = 20; intra-cluster correlation coefficient: ICC = 0.01), the conventional individual-based sample of 201 increased to 240. Considering a 20% loss to follow-up, the final sample size will be 300 participants.

To reduce cross-contamination, clinical procedures are centralized at a reference site (Gestar), and the distribution of printed and digital protocols to the Health Units are restricted until activation. A 2-week operational wash-in with adherence monitoring was implemented.

This project was approved by the Research Ethics Committee of Unoeste – Universidade do Oeste Paulista (CAAE 63,623,922.70000.5515). All patients involved will be informed about the research and sign the informed consent form.

### Interventions

The intervention in this study is designed to manage and mitigate modifiable risk factors associated with spontaneous PTB among pregnant women. It is implemented as a bundle of low-cost measures across different stages of pregnancy, with a focus on three key areas: smoking cessation, maintenance of healthy vaginal microbiota, and management of cervical shortening.

Below is a detailed description of each component of the intervention:

(1) First Trimester (11 to 13 weeks and 6 days): Assessment and Smoking Intervention.
  - **Sociodemographic and Clinical Assessment**: During the first trimester, participants undergo an initial interview to collect personal, social, and clinical information. This includes family and obstetric history, as well as smoking status.
  - **Smoking Cessation Support**: Pregnant women identified as smokers who are willing to quit are provided with a comprehensive smoking cessation program. This includes referral to the city’s smoking prevention program, which is conducted at the Alcohol and Drug Psychosocial Care Center (CAPS-AD).
    - The smoking intervention involves an initial interview and adjuvant treatment with auricular acupuncture. This technique uses pressure points in the ear to help reduce anxiety and withdrawal symptoms associated with smoking cessation.
    - Acupuncture sessions are conducted every 15 days, where seeds are placed on specific points in the ear to stimulate them. Cotton and 70% alcohol are used to clean the ear, an auricular pressure gauge is used to locate the points, and mustard seeds with micropore are placed for acupressure of the selected points. The patients are instructed to stimulate these points themselves three times daily. Auriculotherapy is delivered by a trained and certified professional. Patient engagement is monitored by tracking attendance, with the number of appointments systematically recorded in the medical records.
    - If necessary, group sessions with psychological support are provided at CAPS-AD, and pharmacological treatment, such as nicotine patches, may be used for gradual dose reduction to alleviate withdrawal symptoms.
2. **Second Trimester (20 to 23 weeks and 6 days): Cervical Length Monitoring and Infection Treatment**.
  - **Cervical Length Screening**: Between 20 and 24 weeks of gestation, all participating women undergo a transvaginal ultrasound to measure cervical length, a critical indicator of the risk for PTB.
    - The measurement is taken with the patient in the lithotomy position, using a transvaginal probe to assess the distance between the internal and external orifices of the cervical canal. Women are instructed to empty their bladder before the ultrasound evaluation.
    - Three consecutive measures are taken and the smallest value is considered. If a cervical length of less than 25 mm is detected, the woman is diagnosed with cervical shortening.
    - Women diagnosed with cervical shortening are prescribed 200 mg of vaginal progesterone daily until 36 weeks of gestation, aiming to reduce the risk of PTB. For these women, the measurement is repeated biweekly for follow-up and monitoring treatment adherence.
    - For cases of severe cervical shortening, such as lengths below 15 mm, cerclage – a surgical procedure to reinforce the cervix – is considered, depending on the patient’s clinical history and ongoing evaluations.
  - **Investigation and Treatment of Infections**: During the same trimester, women are also screened for lower genital tract infections using molecular tests for *Chlamydia trachomatis* and *Neisseria gonorrhoeae*. These investigations are performed at this time due to the less friable nature of mid-trimester cervices in comparison to the first trimester of pregnancy.
    - The assessment includes a complete gynecological examination with swabs for Gram staining, vaginal pH measurement, and cytobrush sampling collection for PCR analysis of cervical infection using the automated Cobas® 4800 CT/NG v2.0.
    - Identified infections are treated according to the local clinical protocols to ensure the health of both the mother and the fetus.

**3. Throughout All Trimesters (11 weeks to Term): Vaginal Microbiota Monitoring and Dysbiosis Management.**

- **Quarterly Assessment of Vaginal Microbiota**: During each trimester, vaginal samples are collected from the vaginal wall using sterile swabs to assess eubiosis (the balance of the vaginal microbiota) by bacterioscopy using the Gram staining method according to the criteria set by Nugent et al. [11].
  - The microscopic evaluation aims to identify dysbiosis, such as bacterial vaginosis, candidiasis, or other alterations in the vaginal microbiota. The trained examiner will be blinded to patients’ clinical symptoms and a quality assessment of the readings will be performed by an independent examiner, following the same criteria.
  - Women diagnosed with dysbiosis receive treatment based on the type of condition identified. For bacterial vaginosis, standard antibiotic metronidazole 500 mg orally 12/12 h for 7 days regimens are administered. Recurrent cases are treated with metronidazole gel 0.75% 5 g intravaginally once a day for 5 days or Clindamycin cream 2% (20 mg/g) 5 g intravaginally at bedtime for 7 days, according to the CDC protocol, and the partner is treated with secnidazole orally 1000 mg, single dose.
  - Standard antifungal treatment provided for candidiasis is topic nistatin for seven nights. Recurrent cases are treated with miconazole 2% cream 5 g intravaginally daily for 14 days.
  - Additionally, all participants are educated on proper intimate hygiene practices to help maintain a balanced vaginal environment throughout the pregnancy.

This protocol is conducted alongside standard prenatal care and patients are periodically reminded of their appointments. The study protocol is original and was registered in the Brazilian Clinical Trial Registry (ReBec) on 29 October 2024 (approval pending).

Data regarding the appointments, patients’ compliance with the protocol, and pregnancy outcome will be collected through interviews and medical records by the researchers for subsequent analysis. Data entry will be double checked, periodically updated and securely stored in the institutional repository. Only the researchers will have access to individual-level data, while metadata will be made openly available after publication of the study results.

### Analysis plan

The main outcome of interest is gestational age at birth, based on first-trimester ultrasound, to be compared between the participants of the study and the general population. For the analysis, gestational age will be treated as categorical data (preterm birth vs. term birth). The primary analysis will follow the intention-to-treat principle: participants will be analyzed according to the assignment of their cluster at the time of inclusion. Secondary analyses will consider gestational age at delivery (continuous, in days) and other perinatal outcomes.

Secondary outcomes include the percentage of pregnant women included, the percentage of complete follow-up, percentage of adherence to prescribed treatments, and neonatal outcome (Apgar score, birth weight, and length of stay in the Intensive Care Unit). Descriptive statistics will be performed to analyze clinical, biological, and sociodemographic data of the studied population. Vaginal microbiota status across the trimesters will be compared using the Chi-square test.

Logistic regression will be performed to evaluate the role of the protocol in reducing PTB. To minimize multiplicity in the analysis and interpretation, covariates for adjustment such as parity, maternal age, and socioeconomic status will be included. The primary effect estimate will be obtained from mixed-effects logistic regression models including a random intercept for cluster and fixed effects for period and pre-specified baseline covariates (maternal age, parity, socioeconomic status). Additional per-protocol analyses will be performed as sensitivity analyses and will include participants who received the defined intervention components. Missing data in covariates will be handled using multiple imputations (MICE) under a missing-at-random assumption. The level of significance adopted for the tests used will be 5%, using *Prism* 5.0 (GraphPad Software).

## Discussion

Several factors contribute to prematurity rates at a population level. Despite ongoing efforts to prevent prematurity, protocols for early identification and management of women at risk have not yet been fully defined or widely implemented. This is of particular importance in low‑ and middle‑income countries (LMICs), where the decision- makers are often insufficiently aware of populations health problems or face competing priorities that undermine investment in maternal – fetal health. In addition, population’s lack of awareness of the subject combined with financial and structural constraints further contributes to high PTB rates.

In the context of preterm birth (PTB) prevention, it is crucial to differentiate between merely identifiable risk factors and those that are manageable. Factors such as a personal or family history of PTB are inherently identifiable but cannot be altered. In contrast, modifiable risk factors, including smoking habit, vaginal dysbiosis, and cervical shortening, are more dynamic and can be managed through targeted interventions. These manageable factors are particularly responsive to intervention, meaning that timely and appropriate measures can significantly mitigate their impact on pregnancy outcomes, ultimately reducing the risk of PTB.

While individual interventions addressing these risk factors have been applied in some settings, to our knowledge, this is the first study to implement a low-cost bundle that systematically modulates modifiable risk factors for PTB in a high-prevalence population. Nevertheless, a limitation of our protocol is that the stepped-wedge quasi-experimental design may allow for confounding factors, such as selection bias by voluntary participation and possible cross-cluster contamination, despite the mitigation strategies described. This bundle is applied at multiple stages of pregnancy to address the specific modifiable risk factors of smoking, cervical shortening, and vaginal dysbiosis and infections. By providing tailored support, the bundle aims to lower the incidence of PTB within this cohort. The approach is practical, potentialy scalable to similar populations, and designed to be low-cost, making it suitable for implementation in resource-limited settings.

The implementation of this protocol required overcoming several barriers, such as the slow process of acquiring the ultrasound equipment and strengthening communication between the research team, health professionals, and policymakers. This protocol highlights the importance of implementing active measures to address PTB risk factors in high-prevalence settings and suggests that this strategy could offer meaningful benefits for the prevention of PTB.

## Conclusion

Prevention of syndromes like PTB is difficult to achieve. However, a reduction in rate by targeted and tailored interventions for identifiable and modifiable risk factors is achievable. The effectiveness of such interventions can be twofold: (1) Addressing an existing risk and minimizing its impact on pregnancy outcome, and (2) Addressing unchangeable conditions by providing supportive care and stress relief for subjects who are at risk. The latter can also indirectly control stress hormones and endocrinological challenges that can contribute to preterm birth. Current strategies to understand the pathologic mechanisms and develop effective interventions have not met with great success. We strongly urge a simplified approach to screening high-risk pregnancies focusing on easily identifiable risks and implementing cost-effective interventions that can mitigate PTB.

## Supplementary Material

TiDieR.pdf

FillableSPIRITChecklist2.pdf

## Data Availability

No data was generated for this study. The full SPIRIT checklist is available at https://osf.io/yja4b/, and data will be made available upon reasonable request.

## References

[1] Harrison MS, Goldenberg RL. Global burden of prematurity. Semin Fetal Neonatal Med. 2016;21:74–7. doi: 10.1016/j.siny.2015.12.007 26740166

[2] DataSUS tabnet. [cited 2024 12 out]. Available from: https://datasus.saude.gov.br/informacoes-de-saude-tabnet/

[3] Gravett MG, Menon R, Tribe RM, et al. Assessment of current biomarkers and interventions to identify and treat women at risk of preterm birth. Front Med. 2024;11:1414428. doi: 10.3389/fmed.2024.1414428 PMC11312378 39131090

[4] Soneji S, Beltrán-Sánchez H. Association of maternal cigarette smoking and smoking cessation with preterm birth. JAMA Netw Open. 2019;2:e192514. doi: 10.1001/jamanetworkopen.2019.2514 31002320 PMC6481448

[5] Arcanjelo EDV, Lopes SS, Suliano LC. Tratamento do tabagismo por acupuntura. Rev Bras Terap e Saúde. 2014;4:15–19. doi: 10.7436/rbts-2014.04.02.03

[6] Witkin SS, Moron AF, Ridenhour BJ, et al. Vaginal biomarkers that predict cervical length and dominant bacteria in the vaginal microbiomes of pregnant women. MBio. 2019;10:02242–19. doi: 10.1128/mBio.02242-19 PMC6805993 31641087

[7] Jayaram PM, Mohan MK, Konje J. Bacterial vaginosis in pregnancy - a storm in the cup of tea. Eur J Obstet Gynecol Reprod Biol. 2020;253:220–224. doi: 10.1016/j.ejogrb.2020.08.009 32889328

[8] Pacagnella RC, Silva TV, Cecatti JG, et al. Pessary plus progesterone to prevent preterm birth in women with short cervixes: randomized controlled trial. Obstet Gynecol. 2022;139:41–51. doi: 10.1097/AOG.0000000000004634 34856583

[9] Romero R, Conde-Agudelo A, Da Fonseca E, et al. Vaginal progesterone for preventing preterm birth and adverse perinatal outcomes in singleton gestations with a short cervix: a meta-analysis of individual patient data. Am J Obstet Gynecol. 2018;218:161–180. doi: 10.1016/j.ajog.2017.11.576 29157866 PMC5987201

[10] Peters DH, Adam T, Alonge O, et al. Implementation research: what it is and how to do it. BMJ. 2013;347:f6753. doi: 10.1136/BMJ.f6753 24259324

[11] Nugent RP, Krohn MA, Hillier SL. Reliability of diagnosing bacterial vaginosis is improved by a standardized method of Gram stain interpretation. J Clin Microbiol. 1991;29:297–301. doi: 10.1128/jcm.29.2.297-301.1991 1706728 PMC269757

